# GABAergic basal forebrain projections to the periaqueductal gray promote food consumption, reward and predation

**DOI:** 10.1038/s41598-021-02157-7

**Published:** 2021-11-22

**Authors:** Ciorana Roman-Ortiz, Jessica A. Guevara, Roger L. Clem

**Affiliations:** 1 Nash Family Department of Neuroscience, Friedman Brain Institute, Icahn School of Medicine at Mount Sinai, New York, NY USA; 2 Department of Biological Sciences, St. Francis College, Brooklyn, NY USA

**Keywords:** Neuroscience, Feeding behaviour, Motivation, Neural circuits, Reward

## Abstract

Behaviors central to the procurement and consumption of food are among those most fundamental to survival, but their inappropriate expression can lead to overeating and obesity. Nevertheless, we have a poor understanding of circuits that promote feeding independent of physiological demand. Here we demonstrate that activation of basal forebrain (BF) GABAergic neurons results in consumption of food as well as non-food items in well-fed mice, and performance of fictive eating in the absence of ingestible materials. In addition, stimulation of these cells disrupts defensive threat responses and elicits reward-like motivational effects. Finally, BF GABAergic activity triggers skilled predatory attacks of live prey and prey-like objects, but not social targets. These effects were entirely recapitulated by selective stimulation of BF GABAergic projections to the periaqueductal gray (PAG). Our results outline a potent circuit mechanism for increased feeding through recruitment of distinct but synergistic behaviors, and add to growing evidence that PAG is an important integrator of feeding-related activity.

## Introduction

Maladaptive overeating is one of the main causes of obesity, which in recent decades has doubled in prevalence to an overwhelming 43% of U.S. adults^1^. Binge eating, which is characterized by recurrent periods of eating large amounts of food in the absence of physical hunger, is the most common eating disorder^2,3^. Previous work in rodents has shed considerable light on the circuitry underlying homeostatic food consumption, which involves processing of neural signals for hunger and satiety by the lateral hypothalamus (LH) and arcuate nucleus (ARC)^4–8^. However, we have an incomplete understanding of neural pathways that modulate and/or coordinate more fundamental aspects of the feeding repertoire, the dysregulation of which could lead to food consumption even in the absence of homeostatic drive.

Although they have been primarily implicated in attention, arousal, and sleep/wake regulation^9–12^, recent reports suggest that neuronal populations in the basal forebrain (BF) contribute to food intake^13,14^. For example, activation of cholinergic as well as glutamatergic BF neurons decreases food consumption, whereas activation of GABAergic neurons increases food intake^14–17^. Moreover, BF GABAergic activity increases during naturally occurring food consumption and hunting^17^. Despite the unique potential for BF GABAergic neurons to drive feeding, it remains unclear which aspects of behavior are primarily modulated by these cells or which downstream circuits underlie these responses. Among the areas receiving GABAergic projections from BF is the periaqueductal gray (PAG)^18,19^, a midbrain structure involved in critical survival-based processes including pain, defense, foraging and hunting^20–23^. We therefore sought to establish the impact of BF GABAergic neurons, and their PAG projections, on behaviors central to the procurement and consumption of food. We found that a BF-PAG circuit promotes hunting and instrumental responding for food, as well as its consumption, regardless of caloric value or existing homeostatic demands.

## Results

### Activation of BF^GAD2+^ neurons increases consummatory drive independent of caloric value

**Figure 1:**
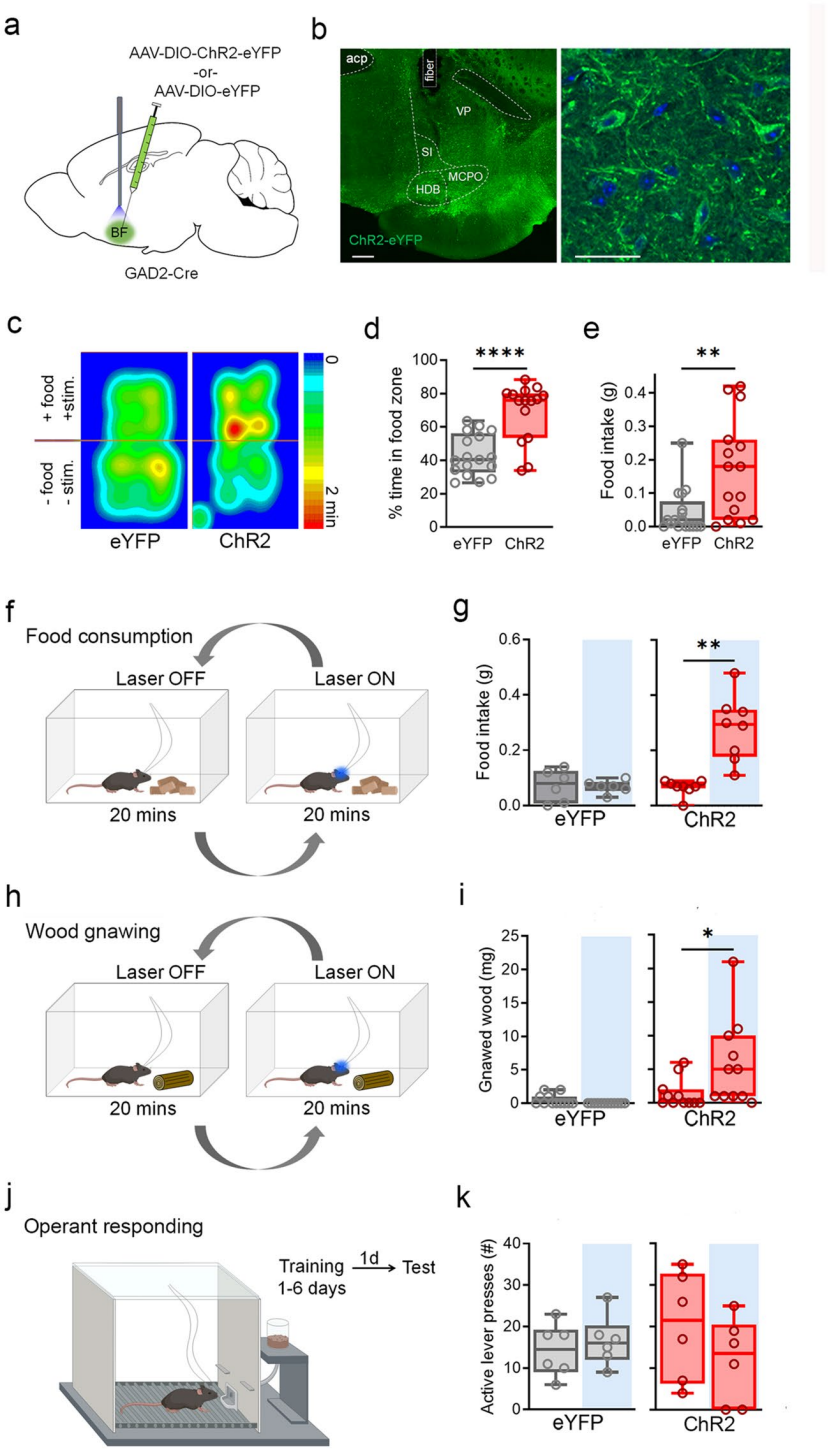
Activation of BF^GAD2+^ neurons increases consummatory drive. (**a**) Stereotaxic targeting of viral vectors and optogenetic stimulation. (**b**) Confocal image of viral expression and optic fiber placement in BF, 200 µm scale bar. Right panel, high power inset of ChR2-expressing neurons in BF, 50 µm scale bar. Blue channel: 4′,6-diamidino-2-phenylindole (DAPI). (**c**) Heatmap of center body location, mean of eYFP and ChR2 groups, during test of food exposure paired with optic stimulation. One side of the arena contained food pellets, whereupon entry triggered optic stimulation. (**d**) Percent time spent in food zone. Mann–Whitney test, *U* = 36, ***p < 0.001. ChR2, n = 15; eYFP, n = 17. (**e**) Food intake, ingested mass. Mann–Whitney test, *U* = 54.5, **p < 0.01. ChR2, n = 15; eYFP n = 17. (**f**) Design for food consumption assay: food biscuits were place at the bottom of the behavioral box and food intake was measured in the presence and absence of laser stimulation, in a counterbalance fashion. (**g**) Food intake, ingested mass during laser^OFF^ and laser^ON^ epochs. Wilcoxon signed rank test, *W* = 36, **p < 0.01. ChR2, n = 8; eYFP n = 6. (**h**) Design for test of wood gnawing behavior, wherein animals were exposed to a willow tree branch with and without laser stimulation, in a counterbalanced fashion. (**i**) Amount of wood removed due to gnawing during laser^ON^ vs laser^OFF^ epochs. ChR2, Wilcoxon signed rank test, *W* = 44, *p < 0.05. ChR2, n = 11; eYFP, n = 12. (**j**) Design of test for operant responding for food. Task entails discrimination between two levers, only one of which upon pressing will result in food delivery. (**k**) Active lever presses during laser^ON^ vs laser^OFF^ epochs. ChR2, n = 6; eYFP, n = 6. Schematics (**a**,**f**,**h**,**j**) were created using BioRender.com and PowerPoint.

In order to examine the behavioral role of BF GABAergic neurons in consummatory behaviors, we employed an optogenetic approach. First, we injected into basal forebrain of GAD2-Cre mice a Cre-dependent adeno-associated virus (AAV) expressing channelrhodopsin (ChR2) fused to an enhanced yellow fluorescent protein (AAV1-Ef1a-DIO-ChR2-eYFP) or an opsin-negative eYFP control vector (AAV1-Ef1a-DIO-eYFP), and implanted optic ferrules directed at the same site (Fig. 1a,b, Supplementary Fig. 1). Because previous studies have reported that photoactivation of BF^GAD2+^ neurons induces food consumption^16,17^, we first tested the effect of photostimulation paired with food availability. The test chamber was divided into two zones, one of which contained food pellets. Upon each entry to the food zone, photostimulation (473 nm; 20 Hz, 10 ms pulses; 5–8 mW) was delivered for 60 s, which resulted in increased time spent in the food zone as well as increased food intake (Fig. 1c–e). Using a different design in which stimulation was delivered in counterbalanced epochs irrespective of subject location, we independently confirmed an increase in feeding upon activation of BF^GAD2+^ neurons (Fig. 1f,g). Given the pronounced effect on food consumption in well-fed animals, we tested whether caloric value was a prerequisite for consumption by exposing the animals to a willow tree branch and measuring the amount of wood removed by gnawing. Animals that received BF^GAD2+^ activation gnawed more wood compared to eYFP control animals (Fig. 1h,i). Thus, activation of BF^GAD2+^ neurons promotes consummatory behavior that is directed at both food and non-food items, and therefore is independent of nutritional characteristics.

Given the apparent modulation of consummatory drive by BF^GAD2+^ neurons, we next examined whether activation of these cells is sufficient to enhance performance in an appetitive operant task, in which animals are required to press an active lever for a food reward on a continuous reinforcement (fixed ratio 1:1, FR1) schedule. Food-restricted mice received daily training sessions lasting 60 min or until they made 30 rewarded lever presses. Once animals met acquisition criteria (30 lever presses within 10 min), they were tested on the following day to determine the effect of BF^GAD2+^ activation on established operant responding. Photostimulation had no discernible effect on lever pressing, indicating that increased consummatory drive did not facilitate a learned food-seeking behavior (Fig. 1j,k).

**Figure 2:**
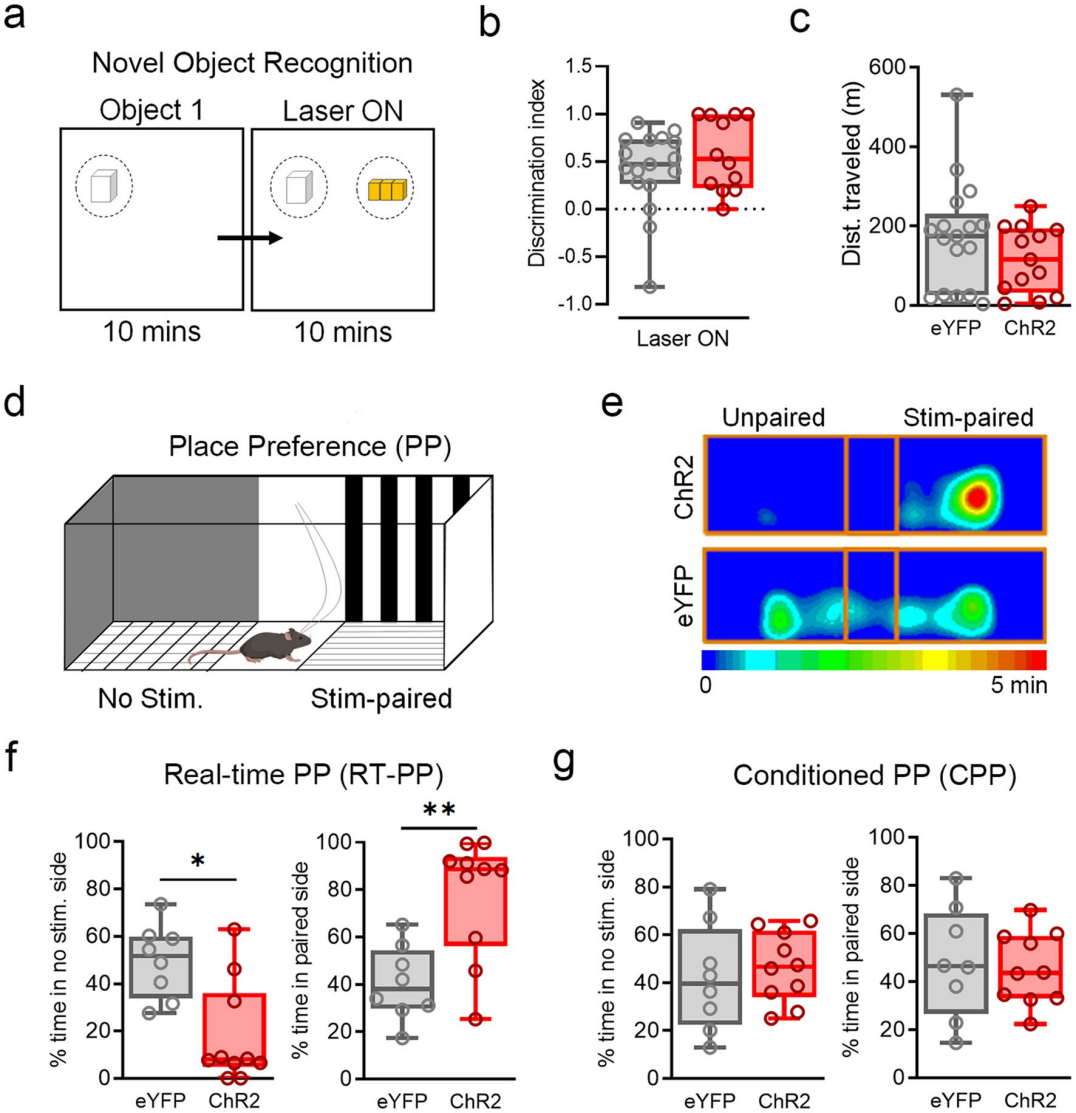
BF^GAD2+^neuron activation does not affect exploration or object recognition and induces reward-like effects. (**a**) Design of novel object recognition test. (**b**) Discrimination index is measured as the time spent exploring the novel object—time spent exploring the familiar object divided by the total time exploring novel object and familiar object. ChR2, n = 12; eYFP, n = 17. (**c**) Total distance traveled during laser^ON^ epoch. ChR2, n = 12; eYFP, n = 17. (**d**) Design for test of real-time and conditioned place preference. (**e**) Heatmap of center body location, of ChR2 and eYFP sample animal. ChR2, n = 10; eYFP, n = 8. (**f**) Real-time place preference, measured on day 1 during photostimulation. Percent time in unpaired side, Mann–Whitney test, *U* = 12, *p < 0.05. Percent time in stimulation paired side, Mann–Whitney test, *U* = 10, **p < 0.01. ChR2, n = 10; eYFP, n = 8. (**g**) Conditioned place preference, measured on day 2 in the absence of photostimulation. ChR2, n = 10; eYFP, n = 8. Schematics (**a**,**d**) were created using BioRender.com and PowerPoint.

To determine whether BF^GAD2+^ activation has gross effects on exploration or cognition, we examined whether photostimulation alters locomotor activity and/or recognition memory in the novel object task. Animals were first familiarized to a novel object and, coinciding with photostimulation, a second novel object was introduced (Fig. 2a). During photostimulation, ChR2- versus eYFP-expressing animals did not differ in distance traveled within the arena (Fig. 2b). Likewise, photostimulation did not disrupt the preference to explore a novel versus familiar object, further indicating that it does not affect ongoing exploration, nor does it impair object perception or recognition memory in this task (Fig. 2c). To ascertain whether BF^GAD2+^ neurons signal emotional valence, we conducted an assay of real-time place preference (RT-PP), in which mice are allowed to freely explore two distinct chambers, one of which is paired with photostimulation (Fig. 2d). Compared to eYFP controls, ChR2-expressing mice spent far greater time exploring the photostimulation-paired chamber, consistent with a real-time preference for BF^GAD2+^ activation (Fig. 2e–g). However, this preference was not retained during subsequent testing in the absence of photostimulation, suggesting that with the given parameters, activation of BF^GAD2+^ cell bodies does support the formation of a contextual reward association.

**Figure 3:**
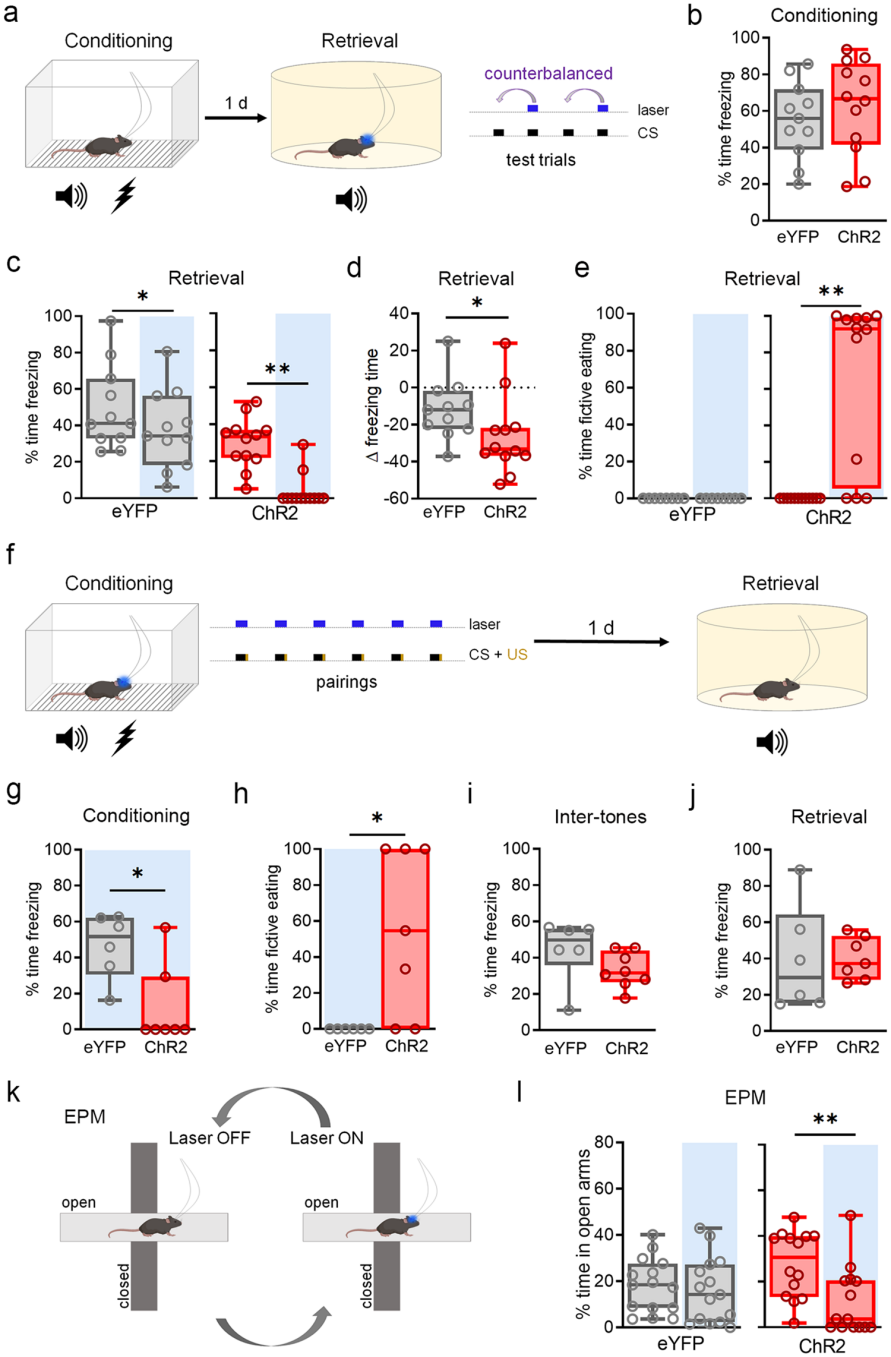
BF^GAD2+^ neuron activation disrupts conditioned fear expression but not learning. (**a**) Design for test of fear memory expression. Following conditioning in context A, animals were placed into a distinct arena (context B) and presented with 4 CS trials, two of which coincided with photostimulation, in a counterbalanced fashion. (**b**) Freezing levels during final two CS trials of conditioning. (**c**) CS-evoked freezing during laser^ON^ vs. laser^OFF^ CS trials. ChR2, Wilcoxon signed rank test, *W* = − 66, **p < 0.01. eYFP, paired t-test, t_10_ = 2.40, *p < 0.05. ChR2, n = 12; eYFP, n = 11. (**d**) Difference in freezing during laser^ON^ vs. laser^OFF^ CS trials. ChR2, n = 12; eYFP, n = 11. (**e**) Fictive eating during laser^ON^ vs. laser^OFF^ CS trials. ChR2, Wilcoxon signed rank test, *W* = 45, **p < 0.01. (**f**) Design for test of fear learning. During fear conditioning (context A) mice were presented with 6 CS-US pairings that coincided with laser stimulation, and on the following day were tested for long-term memory by exposure to 2 CS trials in the absence of laser stimulation in a context distinct from the training arena (context B). (**g**) Freezing levels across all CS trials of conditioning. Mann–Whitney test, *U* = 4, *p < 0.05. ChR2, n = 7; eYFP, n = 6. (**h**) Fictive eating during CS trials. Mann–Whitney test, *U* = 6, *p < 0.05. ChR2, n = 7; eYFP, n = 6. (**i**) Freezing levels during inter-CS intervals. ChR2, n = 7; eYFP, n = 6. (**j**) CS-evoked freezing during the retrieval test. ChR2, n = 7; eYFP, n = 6. (**k**) Design for test of anxiety-like behavior using the elevated plus maze (EPM). (**l**) Percent time spent in open arms during laser^ON^ vs. laser^OFF^ epochs. ChR2, Wilcoxon signed rank test, *W* = − 89, **p < 0.01. ChR2, n = 15; eYFP, n = 15. Schematics (**a**,**f**,**k**) were created using BioRender.com and PowerPoint.

To determine whether BF^GAD2+^ activation influences the expression of a negative valence response, we next examined the impact of photostimulation on cue-elicited freezing following auditory fear conditioning, which entailed 6 presentations of an auditory conditioned stimulus (CS, 2 kHz, 90 dB, 20 s) that co-terminated with an unconditioned stimulus (US, 0.7 mA foot shock, 2 s) (Fig. 3a,b). When activation of BF^GAD2+^ neurons coincided with CS-evoked memory retrieval, freezing was markedly reduced compared to CS-only trials (Fig. 3c,d). Interestingly, during periods of reduced freezing, we noticed that animals were largely engaged in fictive eating behaviors (Fig. 3e), including licking and biting their surroundings, and raising empty paws to the mouth in a conventional feeding movement^24^. Additionally, BF^GAD2+^ activation during baseline period increased fictive eating (Laser^ON^ vs laser^OFF^ epochs; ChR2, Wilcoxon signed rank test, *W* = 45, ** p < 0.01. ChR2, n = 12). In the absence of consumable objects, therefore, BF^GAD2+^ neurons initiate indiscriminate feeding and, surprisingly, imminent threat is not sufficient to suppress them.

To gain more insight into whether BF^GAD2+^ activation alters the perception of negative valence, we conducted photostimulation timed to coincide with each CS-US trial during fear conditioning (Fig. 3f). As observed during fear memory retrieval, BF^GAD2+^ activation largely abolished freezing during conditioning trials and caused animals to instead engage in fictive eating (Fig. 3g,h, Supplementary Fig. 2). However, freezing during interstimulus periods was unaffected (Fig. 3i). Moreover, BF^GAD2+^ activation during conditioning had no effect on CS-evoked freezing measured on the following day (Fig. 3j), suggesting intact processing of negative US valence and successful memory formation. To ascertain whether activation of BF^GAD2+^ neurons might also interfere with anxiety-like responses, we examined the effect of photostimulation on the elevated plus maze (EPM). On the contrary, stimulation of BF^GAD2+^ neurons reduced exploration of the anxiogenic compartments (Fig. 3k,l). Together, these findings suggest that while BF^GAD2+^ activation has reward-like effects (Fig. 2), it interferes with conditioned fear primary by disrupting the execution of defensive responses rather through modulation of stimulus valence or mood.

### Activation of BF^GAD2+^ neurons promotes predatory hunting

**Figure 4:**
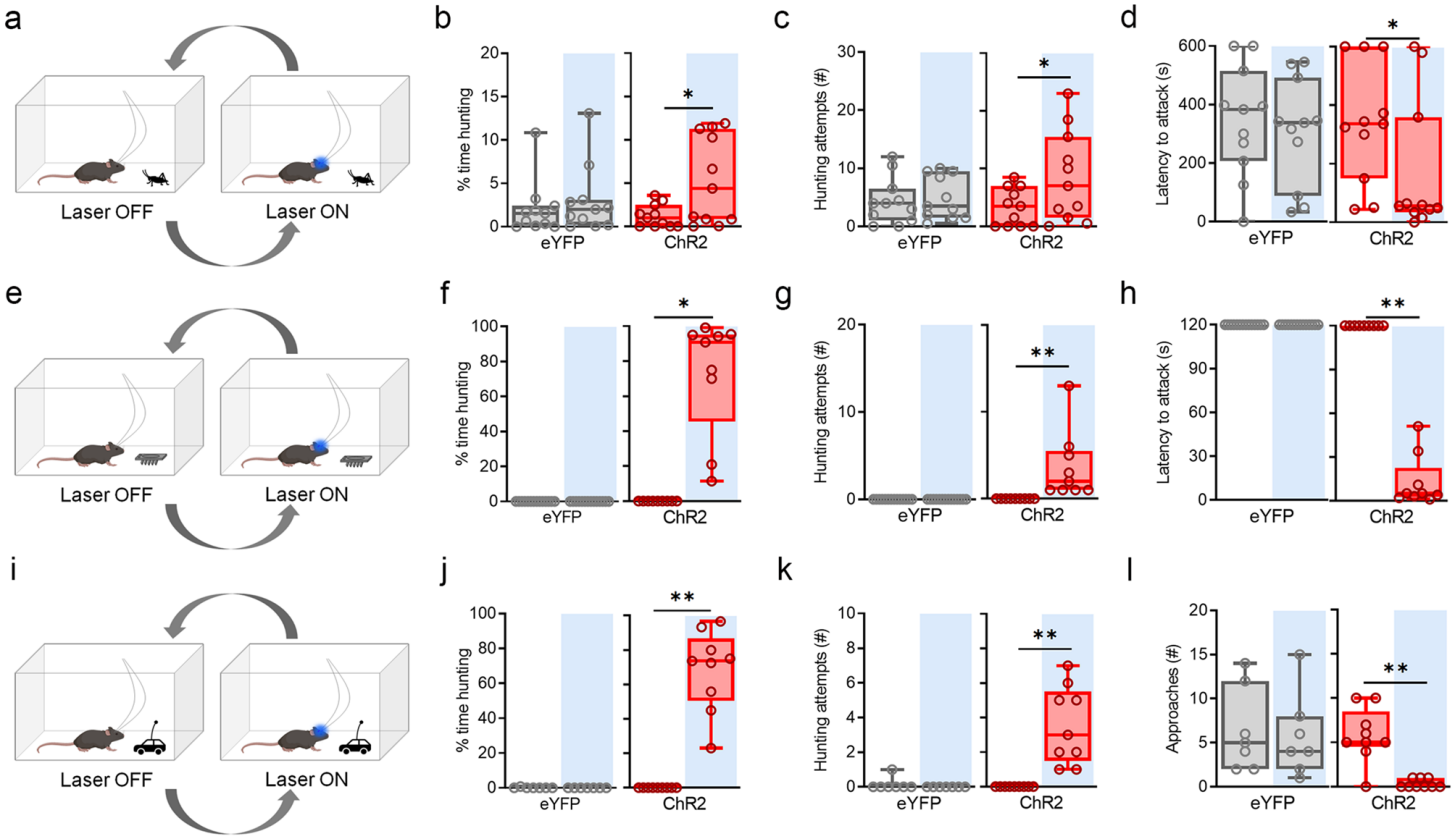
BF^GAD2+^ activation promotes hunting of live and artificial prey. (**a**) Design of live cricket prey hunting assay used in (**b**–**d**). (**b**) Percent time hunting, defined as the time spent pursuing and capturing the cricket. ChR2, Wilcoxon signed rank test, *W* = 43, *p < 0.05. ChR2, n = 11; eYFP, n = 11. (**c**) Number of hunting attempts. ChR2, paired t-test, t_10_ = 2.36, *p < 0.05. ChR2, n = 11, eYFP, n = 11. (**d**) Latency to attack. ChR2, Wilcoxon signed rank test, *W* = − 43, *p < 0.05. (**e**) Design for artificial prey hunting assay used in (**f**–**h**). Artificial prey consisted of a small toy (robobug) that is propelled by vibration. (**f**) Percent time hunting. ChR2, Wilcoxon signed rank test, *W* = 45, **p < 0.01. ChR2, n = 9; eYFP, n = 14. (**g**), Number of hunting attempts. ChR2, Wilcoxon signed rank test, *W* = 45, **p < 0.01. (**h**) Latency to attack. ChR2, Wilcoxon signed rank test, *W* = 45, **p < 0.01. (**i**) Design of the interactive artificial prey assay used in (**j**–**l**). Interactive prey consisted of a toy car remote-controlled by the experimenter. (**j**) Percent time hunting. ChR2, Wilcoxon signed rank test, *W* = 45, **p < 0.01. ChR2, n = 9; eYFP, n = 7. (**k**) Number of hunting attempts. ChR2, Wilcoxon signed rank test, *W* = 45, **p < 0.01. ChR2, n = 9; eYFP, n = 7. (**l**, Number of approaches to the toy car. ChR2, Wilcoxon signed rank test, *W* = 45, **p < 0.01. ChR2, n = 9; eYFP, n = 7. Schematics (**a**,**e**,**i**) were created using BioRender.com and PowerPoint.

Broadly defined, feeding includes all actions integral to the procurement of nutrients. In many mammals, including mice, a critical aspect of feeding involves the pursuit and attack of prey, which is distinct from other forms of aggression. To determine whether BF^GAD2+^ neurons modulate these behaviors, we therefore examined the behavioral effects of photostimulating BF^GAD2+^ neurons in the presence of crickets, which are natural prey of rodents. After habituation to both prey and experimental apparatus, we exposed well-fed mice to a single live cricket and quantified hunting-related behaviors including pursuit, subduction, and biting. During BF^GAD2+^ neuron activation, mice spent more time engaged in these behaviors, made more hunting attempts, and exhibited a decreased latency to initial attack (Fig. 4a–d, Supplementary Movie 1).

Compared to ingestion, hunting involves relatively complex sensorimotor processing, wherein the unique attributes of natural moving prey may be required to elicit and/or sustain predator–prey interactions because they have been optimized by evolution for specific sensory feedback. To test whether these behaviors depend on natural prey characteristics, we therefore employed moving battery-powered toys (robobugs) as artificial prey-like objects (Fig. 4e). Without exception, animals did not engage in hunting when exposed to robobugs under baseline (light off) conditions. However, upon photoactivation of BF^GAD2+^ neurons, we observed an increase in time spent hunting as well as the number of hunting attempts (Fig. 4f,g, Supplementary Movie 2). Indeed, for the majority of ChR2-expressing mice, photostimulation triggered immediate attack (Fig. 4h). To examine responses to an object that is more interactive than the robobug, which makes random movements, we next employed a remote-controlled toy car to which the animals were habituated beforehand to minimize novelty-related fear (Fig. 4i). During the test we purposely pursued the experimental animal with the car in order to force an interaction and, once an attack was initiated, escape was mimicked by retreating away from the subject. Under this scenario, we found that eYFP mice completely abstained from hunting, despite engaging in several investigative approaches (Fig. 4j–l, Supplementary Movie 3). However, during light on ChR2-expressing mice spent far greater time hunting and, rather than investigating the toy, they attacked it.

The above results suggest that BF^GAD2+^ neurons promote hunting regardless of whether the target shares specific attributes of natural prey. Importantly, however, movement is a key factor in prey detection, particularly in a large space such as that employed in our hunting assays. Therefore, to test the impact of movement on hunting of natural and artificial prey, we introduced immobilized crickets or robobugs. In both cases, we found that activation of BF^GAD2+^ neurons failed to increase hunting and, instead, animals defaulted to fictive eating behaviors during photostimulation (Supplementary Fig. 3). These data suggest that movement, and potentially prey interaction, provide feedback essential for the predatory effects of BF^GAD2+^ activation. Although attacks are readily directed at moving objects, however, activation of BF^GAD2+^ neurons did not trigger aggression toward novel conspecifics of either sex or otherwise affect social interaction in the home cage intruder test (Supplementary Fig. 4). Therefore, BF^GAD2+^ neurons regulate a specific form of aggression that is integral to feeding and likely depends on a distinct underlying circuitry.

### BF^GAD2+^ → PAG projections mediate consumption, prey hunting and reward

**Figure 5:**
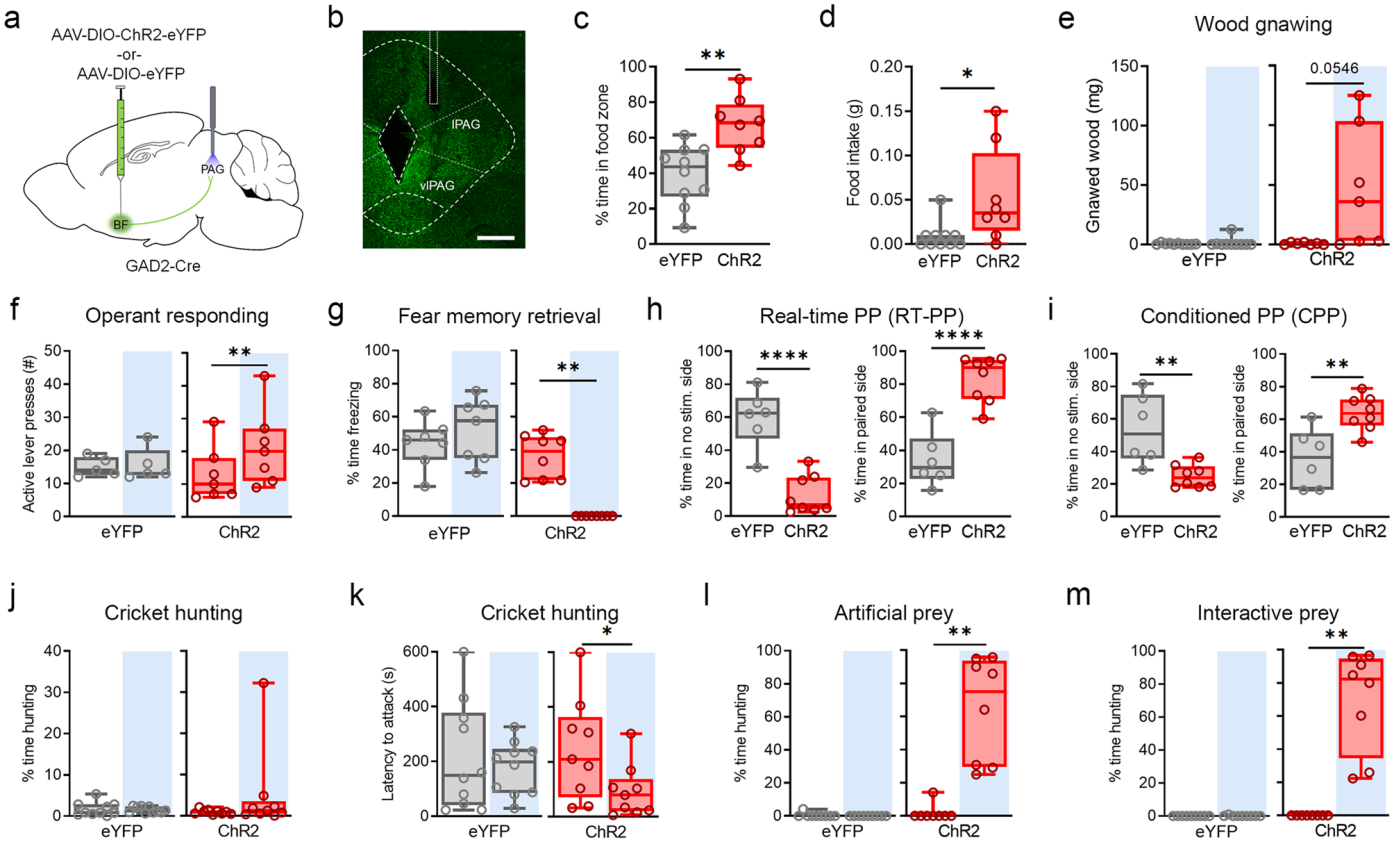
PAG projections mediate BF effects on consumption, reinforcement and predatory hunting. (**a**) Stereotaxic targeting of viral vectors and optogenetic stimulation. (**b**) Confocal image of viral expression and optic fiber placement in BF, 200 µm scale bar**.** (**c**) Percent time spent in food zone during optic stimulation paired with food. Mann–Whitney test, *U* = 14, *p < 0.05. ChR2, n = 8; eYFP, n = 10. (**d**) Food intake, ingested mass. Unpaired t-test, t_16_ = 3.66. **p < 0.01. ChR2, n = 8; eYFP, n = 10. (**e**) Amount of wood removed due to gnawing during laser^ON^ vs laser^OFF^ epochs. ChR2, paired t-test, t_6_ = 2.38. ChR2, n = 7; eYFP, n = 10. (**f**) Active lever presses during test of operant responding for food. ChR2, paired t-test, t_6_ = 3.78, *p < 0.05. ChR2, n = 7; eYFP, n = 5. (**g**) CS-evoked freezing during laser^ON^ vs. laser^OFF^ CS trials, following the experimental design in Fig. 2d. ChR2, Wilcoxon signed rank test, W = − 36, **p < 0.01. ChR2, n = 8; eYFP, n = 7. (**h**) Real-time place preference, measured on day 1 during photostimulation. Percent time in unpaired side, unpaired t-test, t_12_ = 6.03, ****p < 0.0001. Percent time in stimulation paired side, paired t-test, t_12_ = 6.06, ****p < 0.0001. ChR2, n = 8; eYFP, n = 6. (**i**) Conditioned place preference, measured on day 2 in the absence of photostimulation. Percent time in unpaired side, paired t-test, t12 = 3.62, **p < 0.01. Percent time in stimulation paired side, paired t-test, t_12_ = 3.62, **p < 0.01. ChR2, n = 8; eYFP, n = 6. (**j**) Percent time spent hunting in the live cricket assay. ChR2, n = 9; eYFP, n = 10. (**k**) Latency to attack in the live cricket assay. ChR2, paired t-test, t_8_ = 2.82, *p < 0.05. ChR2, n = 9; eYFP, n = 10. (**l**) Percent time hunting in the artificial prey assay. ChR2, Wilcoxon signed rank test, *W* = 36, **p < 0.01. ChR2, n = 8; eYFP, n = 9. (**m**) Percent time hunting in the interactive artificial prey assay. ChR2, Wilcoxon signed rank test, *W* = 36, **p < 0.01. ChR2, n = 8; eYFP, n = 9. Schematic (**a**) was created using BioRender.com and PowerPoint.

Prior anatomical tracing has revealed extensive projections of basal forebrain GABAergic neurons throughout the brain^18,19^. Among their downstream targets are midbrain structures involved in aggression and feeding, such as the hypothalamus and PAG. A well-understood function of GABAergic projections arising from BF nuclei, including the bed nucleus of the stria terminalis (BNST), is the modulation of genetically-defined neuronal populations in the lateral hypothalamus (LH), which promotes food consumption and may be particularly important for hedonic feeding^4,25^. Although LH is also involved in predation, evidence has more extensively implicated the PAG as a critical center for foraging and prey hunting, as well as defending against environmental threats encountered during these risky activities^22,23,26,27^. Examination of PAG confirmed the presence of dense projections from BF^GAD2+^ neurons that are particularly concentrated in the lateral and ventrolateral divisions (Fig. 5a,b), which have been specifically associated with predatory behavior^28^. We therefore utilized projection-specific photoexcitation to test whether this pathway mediates effects of BF^GAD2+^ activity on prey hunting as well as other feeding behaviors.

Following infusion of an AAV-DIO-ChR2 vector into the basal forebrain of GAD2-Cre mice, we implanted an optic fiber directed at the PAG to enable selective stimulation of BF^GAD2+^ → PAG projections (Fig. 5a,b, Supplementary Fig. 5). Photostimulation (473 nm, 20 Hz, 10 ms pulses, 5–8 mW) of this pathway was sufficient to increase consumption of food pellets as well as time spent in the food-paired zone of a two-chamber arena (Fig. 5c,d). In addition, stimulation of ChR2-expressing mice but not eYFP controls promoted wood gnawing (Fig. 5e). Interestingly, activation of BF^GAD2+^ →  PAG pathway also increased lever presses in the appetitive operant task, indicating that unlike stimulation of BF cell bodies (Fig. 1k), selective activation of this pathway facilitates food seeking behavior (Fig. 5f). This may indicate that modulation of other downstream targets of BF opposes or otherwise weakens the positive motivational effect of BF^GAD2+^ →  PAG stimulation.

Following auditory fear conditioning, activation of BF^GAD2+^  →  projections recapitulated the impairment of CS-evoked freezing observed during cell body illumination (Fig. 5g) and this was likewise accompanied by the expression of fictive eating behaviors (Supplementary Fig. 6). In the test of place preference, photoexcitation resulted in a real-time preference for stimulation-paired chamber, consistent with reward-like reinforcement (Fig. 5h). In addition, in contrast to stimulation of BF^GAD2+^ cell bodies (Fig. 2g), BF^GAD2+^ → PAG activation resulted in robust conditioned place preference (CPP), which was expressed on the following day in the absence of photostimulation (Fig. 5i). These results indicate that BF^GAD2+^ → PAG projections can completely account for the consummatory effects of BF^GAD2+^ neurons and their selective activation is associated with stronger motivational effects in both Pavlovian and instrumental tasks.

Using an experimental design identical to that employed during BF^GAD2+^ cell body illumination, we then examined the potential for BF^GAD2+^ → PAG projections to modulate hunting of natural and artificial prey. During exposure to live crickets, photostimulation had no effect on hunting time, possibly because hunting attempts in stimulated animals were highly successful, but it decreased the latency to attack (Fig. 5j,k). Similar to cell body illumination, BF^GAD2+^  → PAG activation also promoted hunting of moving artificial prey (robobugs) as well as an interactive toy car in animals that were not otherwise inclined to hunt these objects (Fig. 5l,m). Therefore, a BF GABAergic pathway targeting the PAG supports fundamental components of the feeding and promotes expression of stereotyped responses regardless of whether the target of these behaviors is suitable for consumption, let alone palatable.

## Discussion

Recent studies have shown that BF cell types modulate feeding behaviors^14–17^, and that GABAergic subtypes, in particular, promote the consumption of palatable food. Here we demonstrate that consistent with these reports activation of BF GABAergic neurons increases consumption but, importantly, this behavior is independent of the nutritional value. Indeed, in the absence of ingestible material, stimulated animals engage in non-productive fictive eating. Additionally, BF GABAergic activity induces predatory hunting of natural prey as well as prey-like electronic devices. These effects are recapitulated by selective stimulation of BF → PAG GABAergic projections, and accompanied by the loss of defensive threat responding, suggestive of a powerful motivational state that overrides competing survival-based demands.

Modulation of feeding in well-fed animals establishes a functional parallel between BF GABAergic neurons and GABAergic projection cells of the LH and CeA^24,29^. Induction of real-time and conditioned place preference suggests that stimulation of BF neurons and their GABAergic projections exerts a positive motivational effect, which may in part explain the lack of discrimination between food and non-food items. On the other hand, engagement in fictive eating during BF stimulation suggests the potential recruitment of anatomical pathways impinging on the parvocellular reticular formation (PCRt), a medullary premotor structure that controls oromotor and forelimb movements^30,31^. While the PCRt is a downstream target of the BF^19^, PAG neurons primarily project to and modulate the mesencephalic locomotor region^32^, although sparse projections to the PCRt have been reported^33^. Involvement of PCRt in BF → PAG effects is therefore likely to require an intermediary relay from the PAG. One possibility is the CeA, which receives extensive PAG projections and in turn modulates orofacial movements via monosynaptic connections with PCRt^18,24^.

In addition to promoting consumption, we found that stimulation of BF GABAergic neurons promoted skilled predatory hunting, as well as sustained attack of prey-like electronic devices. However, this was not observed in the case of immobilized prey, indicating that in addition to BF activity predation is facilitated by integration of specific sensory feedback. In addition, while animals readily engaged in hunting of moving artificial prey during photostimulation, they abstained from attacking a novel conspecific animal, indicating a selective involvement of BF^GAD2+^ neurons in predatory but not social aggression. This further implies that perceptual discrimination is a prerequisite for target engagement upon activation of BF → PAG GABAergic projections.

A particularly intriguing effect of BF GABAergic activity was the disruption of conditioned fear responses. Given reward-like effects of BF GABAergic neurons, we hypothesized that activation of these cells might influence the processing of negative valence during memory retrieval by neutralizing or otherwise overriding the aversive CS. However, manipulation of BF GABAergic neurons during fear conditioning did not affect formation of CS-US associations, nor did it have any effect on freezing occurring outside of stimulation epochs. This likely indicates that BF GABAergic cells do not directly modulate pathways that signal aversive valence, but instead disrupt the execution of defensive responses. However, it is noteworthy that photostimulation did not impair exploratory locomotion, novel object recognition, operant responding or social interaction, which were successfully executed despite increased consummatory drive. Interestingly, threat defense plays an important role in constraining predator–prey interactions, in which target prey represent both a source of sustenance as well as potential harm^26,34^. Because the PAG plays an integral role in both predation and defensive freezing^22,23,32,35^ , further investigation of BF → PAG circuits may provide novel insights into how competing survival-based drives interact to exert moment-to-moment control over behavior.

To a surprising degree, our data implicate GABAergic BF → PAG projections in several distinct components of feeding, including consumption, reward and hunting. This stands in contrast to a previous study in which CeA → PAG projections were found to exert selective control over prey pursuit, but not consumption^24^. This discrepancy may be attributable to differences in the postsynaptic targeting of BF and CeA pathways, particularly given that PAG has been independently implicated in food consumption^27^. Evidence suggests that a common downstream effector of GABAergic populations in the BNST, and LH in consummatory behavior may be suppression of GABAergic neurons in the ventrolateral PAG^26,27^. Indeed, direct suppression of ventrolateral PAG activity leads to food intake as well as long-term weight gain, while inactivation of lateral PAG induces prey hunting^24,26,27,36,37^. Meanwhile, afferents from the CeA and medial preoptic area (MPA) have been shown to modulate defensive freezing and predation, respectively^32,38^. Understanding how PAG encodes these fundamental and oftentimes conflicting behavioral responses will require a more detailed understanding of its intrinsic circuitry and afferent connectivity.

## Methods

### Animals

Adult GAD2-IRES-Cre male and female mice (P60-90) were maintained on a 12 h light–dark cycle with ad libitum access to food and water. Transgenic mice GAD2-IRES-Cre (Stock No. 019022) were acquired from The Jackson Laboratory (Bar Harbor, ME, USA). All experimental procedures were approved by the Institutional Animal Care and Use Committee at the Icahn School of Medicine at Mount Sinai, which is accredited by the Association for the Assessment and Accreditation of Laboratory Animal Care. All experiments were performed in accordance with relevant guidelines and regulations. All experiments are reported in accordance with the ARRIVE guidelines.

### Stereotaxic virus injection and optic fiber implantation

Mice received bilateral infusion of AAV1-EF1a-DIO-hChR2 (H134R)-eYFP (Addgene 20298) or AAV1-Ef1a-DIO-eYFP (Addgene 27056) into basal forebrain (BF, encompassing ventral pallidum, substantia innominata and horizontal diagonal band; bregma coordinates AP + 0.6, ML ± 1.6, DV − 5.1 to 3). Optic fibers ferrules (200 μm diameter, Thorlabs) were placed over BF (AP + 0.6, ML ± 1.6, DV − 5.1) or PAG (AP − 4.36, ML ± 0.42, DV − 3.30) and fixed with acrylic cement. Mice were allowed to recover for 7 days after surgery and habituated to experimenter handling for 3 consecutive days before behavioral testing.

### Behavioral testing

All experiments were carried out while animals were tethered to a patch cord to allow for laser light delivery. The order of light off and light on epochs was counterbalanced across all groups and experiments to control for any ordering effects of photostimulation. All arenas were cleaned with Rescue™ disinfectant (Oakville, ON, USA) after each animal to eliminate any odor cues. Video recording of each test was performed using Any-maze behavioral tracking software (Version 4.99, Stoelting Co., Wood Dale, IL, USA) unless otherwise specified in the sections below. Hunting behaviors as well as social interaction was manually scored by a trained observer naïve to experimental groups.

### Food paired photostimulation

For measurement of food consumption, mice were pre-exposed to dustless food pellets (45 mg, BIO-SERV, Flemingtown, NJ, USA) in their home cage for at least 3 days prior to behavioral testing. Fed (ad libitum) mice were placed in a behavioral box (L: 29.21 cm × W: 19.05 cm × H: 16.51 cm) divided into two zones; a food zone (with a container of food pellets) and a no food zone (which was empty). The food zone was paired with light stimulation (473 nm; 20 Hz, 10 ms pulses; 5–8 mW) for 60 s upon entry. Animals were allowed to move between the two zones for 20 min, and time spent in each zone was quantified using Any-maze behavioral tracking software (Version 4.99, Stoelting Co., Wood Dale, IL, USA).

### Food consumption assay

Fed (ad libitum) mice were placed in a cage (L: 39.1 cm × W:19.9 cm × H:16 cm) that had regular chow biscuits (PicoLab^®^ Rodent Diet 20-5053, LabDiet, St. Louis, MO, USA) scattered across the floor. Food consumption was assessed for two epochs that lasted 20 min each. Laser stimulation (473 nm; 20 Hz, 10 ms pulses; 5–8 mW) was paired with one of the two epochs, with the order of photostimulation being counterbalanced.

### Wood gnawing test

For the wood gnawing assay, we placed a willow tree branch (7.62 cm) in a clean empty behavioral arena (L: 29.21 cm × W: 19.05 cm × H: 16.51 cm) and then placed the mice in the arena. The test consisted of two epochs (20 min each), one of which was paired with laser stimulation (473 nm; 20 Hz, 10 ms pulses; 5–8 mW) in a counterbalanced order. Between each epoch the cage was cleaned with Rescue™ disinfectant (Oakville, ON, USA), and the weight of the branch was obtained.

### Cricket hunting

In order to familiarize the experimental mice with crickets, we placed the mice in a behavioral box with 3 crickets for 30 min on each of 3 days prior to the hunting assay. On the day of the assay, fed (ad libitum) or fasted (12 h, dark cycle) mice were placed in a test arena (L: 40 cm × W: 20 cm × H: 25 cm) allowed to habituate for 10 min. The cage was then cleaned with Rescue™ disinfectant (Oakville, ON, USA), and to start each trial the mouse was placed in one corner of the cage and the cricket (juvenile, medium-sized) was released in the opposite corner. Each trial lasted 10 min and involved a fresh (unharmed) cricket. We performed four trials for each mouse, alternating between laser off and laser on (473 nm; 20 Hz, 10 ms pulses; 5–8 mW) for each trial, with the order of photostimulation being counterbalanced across animals. For the static prey assay dead crickets were used in place of live ones. The trial videos were manually scored for hunting behaviors by a trained observer blind to experimental groups.

### Artificial prey hunting

Mice were placed in a clean empty cage (L: 39.1 cm × W:19.9 cm × H:16 cm) with artificial moving prey (miniature (3.81 cm) battery-powered toy, HEXBUG^®^ Nano, Amazon.com) for two epochs that lasted 2 min each, similar to the hunting assay used in (Han, Tellez et al. 2017). One day prior to testing, mice were habituated to the HEXBUG^®^ for 30 min. To assay hunting of the static toy, we used the same design with the exception that the HEXBUG^®^ was switched off and therefore not moving. Laser stimulation (473 nm; 20 Hz, 10 ms pulses; 5–8 mW) was paired with one of the two epochs, with the order of photostimulation being counterbalanced. Hunting behaviors were manually scored by a trained observer naive to experimental conditions.

### Interactive toy test

To facilitate interaction between the experimental mice and prospective artificial prey we used a remote-controlled toy car (Arris Mini RC Car, Amazon.com, L: 6.7 cm × W: 2 cm × H: 2.8 cm) that was guided by the experimenter. One day prior to the test mice were first habituated to the toy for 30 min in the test arena (L: 40 cm × W: 20 cm × H: 25 cm). The test consisted of two epochs (2 min each), alternating between laser off and laser on (473 nm; 20 Hz, 10 ms pulses; 5–8 mW), with the order of photostimulation being counterbalanced. Hunting behaviors were manually scored by a trained observer blind to experimental conditions.

### Real-time and conditioned place preference

Real-time place preference (RT-PP) was performed in a rectangular box consisting of three compartments: two side chambers (L: 28 cm × W: 24 cm each) connected by a center chamber (L: 11.5 cm × W:24 cm). Each compartment has distinct tactile (small grid vs. bars vs. large grid flooring) and visual (grey vs. black vs. stripped walls) cues to create different environments. One day prior to testing, mice were allowed to explore the entire apparatus for 30 min (habituation). On the day of the test, one side of the chamber was paired with laser light (473 nm; 20 Hz, 10 ms pulses; 5–8 mW) beginning upon entry. The test lasted 20 min. Chamber pairings were counterbalanced across animals. On the next day, during the test of conditioned place preference (CPP; 5 min), mice were again allowed to freely explore the entire apparatus with no laser light presented. Mouse location was tracked and quantified with Any-maze behavioral tracking software (Version 4.99, Stoelting Co., Wood Dale, IL, USA).

### Operant appetitive task

Mice were allowed to acclimate to the behavior room for 30 min before each session. Prior to the first training session the animals were fasted (12 h, dark cycle) and subsequently maintained on a food restricted diet across training. Operant food reinforcement was performed in standard operant chambers (Model MED-307W-D1; Med Associates, Fairfax, VT, USA) equipped with 2 retractable levers (active and inactive). The active lever was defined as the lever that upon pressing results in delivery of a chocolate flavored pellet (45 mg, BIO-SERV, Flemingtown, NJ, USA) in a fixed ratio 1 (FR1) schedule (one-to-one), while inactive lever presses resulted in no programmed reward. Active lever identity was counter-balanced across all animals. Each training session was terminated when the mice reached a maximum of 30 pellets or when the training session reached 60 min, whichever occurred first. The mice were trained for one session per day until they achieved 30 active lever presses during the first 10 min of the sessions (~ 3–5 sessions). Once the animals met acquisition criteria, a test lasting 20 min, with no maximum reward, was administered on the following day. The test had two epochs: one with laser off and one with laser on (473 nm; 20 Hz, 10 ms pulses; 5–8 mW), with the order of photostimulation being counterbalanced. Lever presses (active and inactive) were obtained using Med-PC V Software Suite (Fairfax, VT, USA).

### Novel object recognition

The test consisted of three phases; habituation (5 min), Object 1 exploration (familiarization, 10 min), and object 1 vs object 2 (familiar object vs. novel object, 10 min). During the habituation phase the mice were able to freely explore the empty arena (42 cm × W: 42 cm × H: 30 cm), then at the beginning of the familiarization phase a small object was placed into the arena, and during the last phase a second distinct object was introduced. The objects used for the test varied in texture and color to allow for differentiation. Laser light (473 nm; 20 Hz, 10 ms pulses; 5–8 mW) was only present in the last phase (familiar object vs. novel object), and object exploration time and distance traveled was quantified with Any-maze behavioral tracking software (Version 4.99, Stoelting Co., Wood Dale, IL, USA). The discrimination index was calculated as follows; time spent exploring novel object—time spent exploring familiar object/ total time spent exploring novel and familiar objects.

### Fear conditioning

Fear conditioning and memory retrieval were performed as described in (Cummings and Clem 2020). Briefly, mice underwent fear conditioning consisting of six pairings of an auditory tone (2 kHz, 90 dB, 20 s) with a co-terminating foot shock (0.7 mA, 2 s). The test of fear memory retrieval consisted of four tone presentations in a different context (Context B), and three laser light presentations (473 nm; 20 Hz, 10 ms pulses; 5–8 mW for 20 s). Two of the tone presentations were paired with laser light in an alternating fashion. For optogenetic stimulation during fear conditioning, we administered laser light (473 nm; 20 Hz, 10 ms pulses; 5–8 mW for 20 s) overlapping with all CS-US pairings during conditioning to assess whether optogenetic manipulations disrupt fear learning. To measure retention of fear learning, we examined the response to 2 CS presentations in the absence of laser light stimulation. Freezing and fictive eating was manually scored by a trained observer blind to experimental conditions from videos recorded using Video Freeze^®^ software (Med Associates, Fairfax, VT, USA).

### Elevated plus maze

Mice were placed in the center of the elevated plus maze (Model ENV-560A, Med Associates, Fairfax, VT, USA), where exploration of the maze was tracked and quantify with Any-maze behavioral tracking software (Version 4.99, Stoelting Co., Wood Dale, IL, USA). The test lasted 5 min and was divided into three epochs (100 s each), from which one was paired with laser light (473 nm; 20 Hz, 10 ms pulses, 5–8 mW) in a counterbalanced fashion across all animals.

### Intruder test

Experimental mice were allowed to freely explore a new home cage (L: 39.1 cm × W:19.9 cm × H:16 cm) for 5 min (habituation), before introduction of an age-matched novel conspecific (female or male). Social interaction was then monitored during two contiguous epochs (lasting 3 min each), one of which was paired with photostimulation (473 nm; 20 Hz, 10 ms pulses; 5–8 mW, 3 min) in a counterbalanced order. Time interacting was manually scored by a trained observer blind to experimental conditions.

### Data analysis

Before inclusion of individual animals, both viral expression and fiber placement within the BF were histologically confirmed. Normality was assessed using a Shapiro–Wilk test of individual group data as well as residual values of parametric comparisons, where applicable. Parametric comparisons (i.e. paired or unpaired t-tests) were used only when normality was supported under both conditions. Otherwise, non-parametric comparisons (i.e. Mann–Whitney, Wilcoxon signed ranked test) were used. Because data were generally non-Gaussian, we did not test for interactions between independent variables (e.g. using two-way ANOVA). Statistical analysis and graphing were performed in Prism 9 (Graphpad; La Jolla, CA, USA). Statistical significance was set at P < 0.05 for all parametric and non-parametric tests and only comparisons with P < 0.05 are explicitly reported. Detailed statistics are provided in figure legends. Figure data are expressed in the form of box and whisker plots overlayed with individual data points. Box and whisker plots are constructed as follows: line = median; boxes = 25th–75th percentiles; whiskers = minimum and maximum values. Behavioral diagrams were created using BioRender.

## Supplementary Information

Supplementary Figures.

Supplementary Video S1.

Supplementary Video S2.

Supplementary Video S3.
