# Supplementary material for: GABAergic basal forebrain projections to the periaqueductal gray promote food consumption, reward and predation
Source: Sci Rep. 2021 Nov 22;11:22638. doi: 10.1038/s41598-021-02157-7 (PMC8608827; doi:10.1038/s41598-021-02157-7)
Supplement: Supplementary file 1 — Supplementary Figures. [file 41598_2021_2157_MOESM1_ESM.pdf]

SUPPLEMENTARY FIGURE LEGENDS

Supplementary Figure 1. Optic ferrule placement for stimulation of BF^{GAD2+} cell bodies. Schematic representation of optic ferrule tip placement in BF. The circles represent individual animals ChR2 (red), n = 27; eYFP (grey), n = 30.

Supplementary Figure 2. Effects of BF^{GAD2+} photostimulation during fear acquisition. Freezing levels (context A) of each CS-US presentations with laser stimulation during fear conditioning. ChR2, n = 7; eYFP, n = 6.

Supplementary Figure 3. Animals default to fictive eating during BF^{GAD2+} stimulation in the presence of immobilized prey.

a, Percent time attacking an immobilized (dead) cricket during $laser^{ON}$ vs. $laser^{OFF}$ epochs. ChR2, n = 10; eYFP, n = 11. b, Number of attacks of an immobilized cricket during $laser^{ON}$ vs. $laser^{OFF}$ epochs. ChR2, n = 10; eYFP, n = 11. **c**, Percent time spent fictive eating during $laser^{ON}$ vs. $laser^{OFF}$ epochs of the immobilized cricket assay. ChR2, Wilcoxon signed rank test, $W = 21$, * $p < 0.05$. ChR2, n = 10; eYFP, n = 11. **d**, Percent time attacking an immobile artificial prey (robobug) during $laser^{ON}$ vs. $laser^{OFF}$ epochs. ChR2, n = 11; eYFP, n = 11. **e**, Number of attacks of an immobile artificial prey (robobug) during $laser^{ON}$ vs. $laser^{OFF}$ epochs. ChR2, n = 11; eYFP, n = 11. **f**, Percent time spent fictive eating during $laser^{ON}$ vs. $laser^{OFF}$ epochs of the immobile artificial prey assay. ChR2, Wilcoxon signed rank test, $W = 21$, * $p < 0.05$.

Supplementary Figure 4. Social interaction is not affected by BF^{GAD2+} stimulation.

a, Diagram depicting intruder test design. Social interaction time includes nose-to-nose, nose-to-rear, grooming intruder and mounting. **b**, Percent time spent interacting with a female intruder. Female Intruder: Females: ChR2, n = 6; eYFP, n= 8. Males: ChR2, n = 6; eYFP, n = 6.

c, Percent time spent interacting with a male intruder. Male intruder: Females: ChR2, n = 6; eYFP, n= 8; Males: ChR2, n = 6; eYFP, n = 6. Schematics (**a**) were created using BioRender and PowerPoint.

Supplementary Figure 5. BF^{GAD2+} →PAG optic ferule placement for pathway activation. Schematic representation of optic ferule end placement in PAG. The circles represent individual animals ChR2 (red), n = 12; eYFP (grey), n = 10.

Supplementary Figure 6. Expression of conditioning-related freezing and fictive eating for BF^{GAD2+}→PAG optogenetic experiment. Related to Figure 5. **a**, Freezing levels during final two CS trials of conditioning. ChR2, n = 8; eYFP, n =7. b, Fictive eating during laser^{ON} vs. laser^{OFF} CS trials. ChR2, Wilcoxon signed rank test, $W = 28$, * $p < 0.05$. ChR2, n = 8; eYFP, n = 7.

Supplementary Movie 1. BF $^{GAD2+}$ activation induces cricket hunting. Related to Figure 3. Optogenetic activation of BF $^{GAD2+}$ neurons caused mice to engage in predatory behaviors toward a live cricket.

Supplementary Movie 2. BF^{GAD2+} activation induces predatory hunting of an artificial prey. Related to Figure 3. Optogenetic activation of BF^{GAD2+} neurons caused mice to engage in predator-like attacks towards an artificial prey.

Supplementary Movie 3. BF^{GAD2+} activation induces predatory hunting of an interactive prey. Related to Figure 3. Optogenetic activation of BF^{GAD2+} neurons caused mice to engage in predator-like attacks towards an interactive artificial prey.

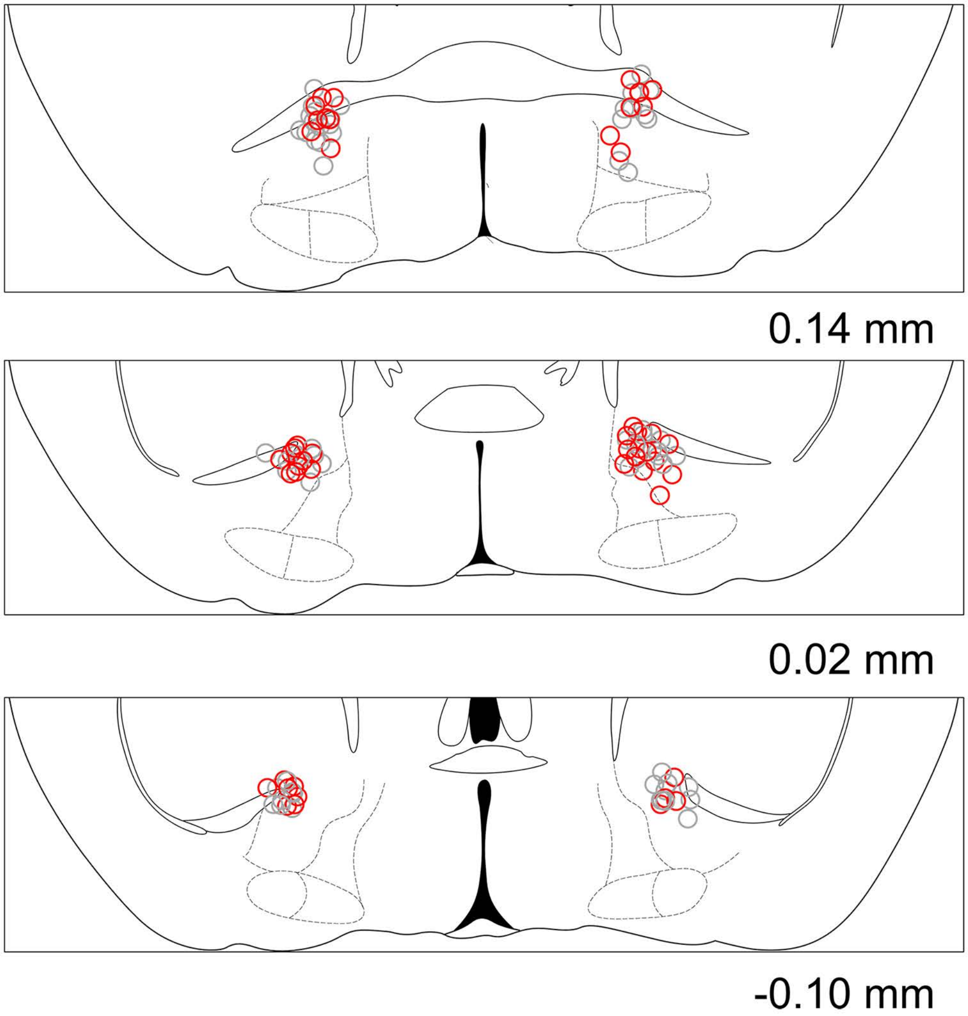

Supplementary Figure 1. Optic ferrule placement for stimulation of BF^{GAD2+} cell bodies. Schematic representation of optic ferrule tip placement in BF. The circles represent individual animals ChR2 (red), n = 27; eYFP (grey), n = 30.

a

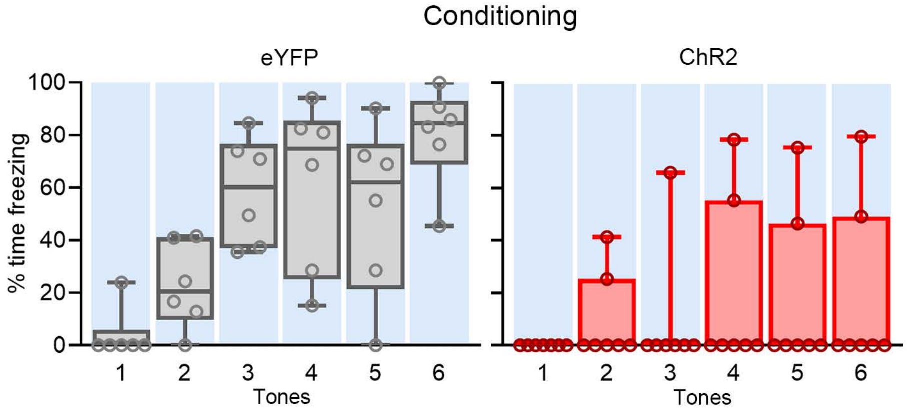

Supplementary Figure 2. Effects of BF^{GAD2+} photostimulation during fear acquisition.
Freezing levels (context A) of each CS-US presentations with laser stimulation during fear conditioning. ChR2, n = 7; eYFP, n = 6.

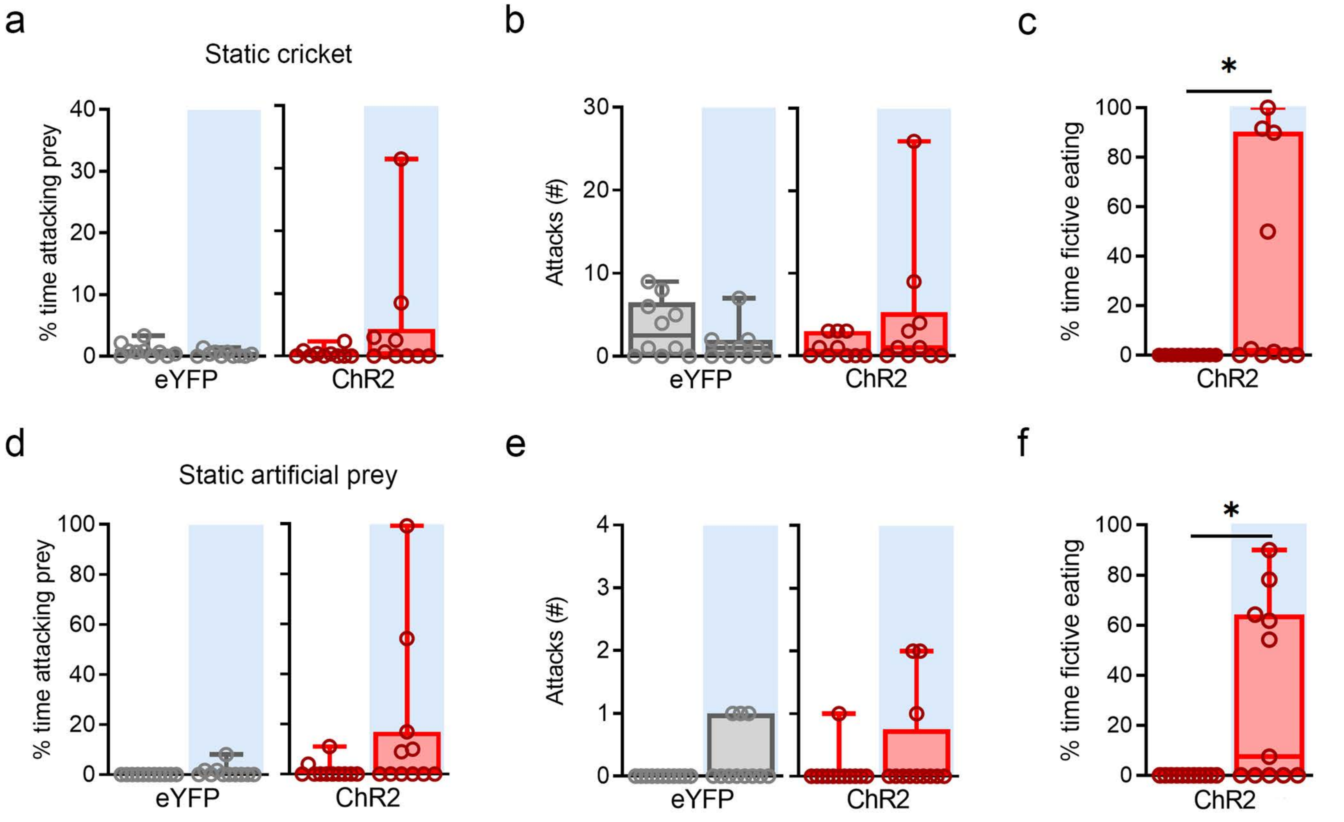

Supplementary Figure 3. Animals default to fictive eating during BF^{GAD2+} stimulation in the presence of immobilized prey.
a, Percent time attacking an immobilized (dead) cricket during laser^{ON} vs. laser^{OFF} epochs. ChR2, n = 10; eYFP, n = 11. b, Number of attacks of an immobilized cricket during laser^{ON} vs. laser^{OFF} epochs. ChR2, n = 10; eYFP, n = 11. **c**, Percent time spent fictive eating during laser^{ON} vs. laser^{OFF} epochs of the immobilized cricket assay. ChR2, Wilcoxon signed rank test, W = 21, * p < 0.05. ChR2, n = 10; eYFP, n = 11. **d**, Percent time attacking an immobile artificial prey (robobug) during laser^{ON} vs. laser^{OFF} epochs. ChR2, n = 11; eYFP, n = 11. **e**, Number of attacks of an immobile artificial prey (robobug) during laser^{ON} vs. laser^{OFF} epochs. ChR2, n = 11; eYFP, n = 11. **f**, Percent time spent fictive eating during laser^{ON} vs. laser^{OFF} epochs of the immobile artificial prey assay. ChR2, Wilcoxon signed rank test, W = 21, * p < 0.05.

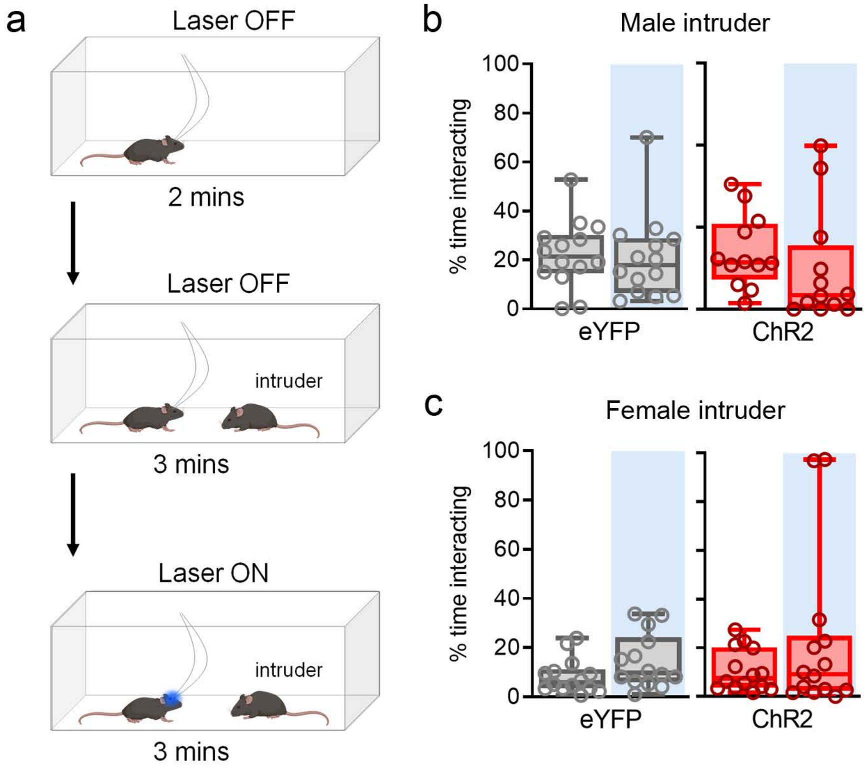

Supplementary Figure 4. Social interaction is not affected by BF^{GAD2+} stimulation.
a, Diagram depicting intruder test design. Social interaction time includes nose-to-nose, nose-to-rear, grooming intruder and mounting. **b**, Percent time spent interacting with a female intruder. Female Intruder: Females: ChR2, n = 6; eYFP, n= 8. Males: ChR2, n = 6; eYFP, n = 6. **c**, Percent time spent interacting with a male intruder. Male intruder: Females: ChR2, n = 6; eYFP, n= 8; Males: ChR2, n = 6; eYFP, n = 6. Schematics (**a**) were created using BioRender and PowerPoint.

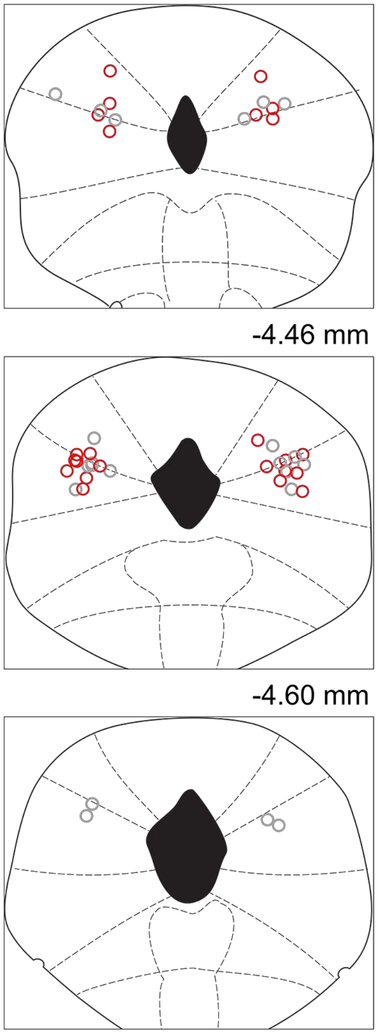

-4.46 mm

-4.60 mm

-4.72 mm

Supplementary Figure 5. BF^{GAD2+} →PAG optic ferule placement for pathway activation.
Schematic representation of optic ferule end placement in PAG. The circles represent individual animals ChR2 (red), n = 12; eYFP (grey), n = 10.

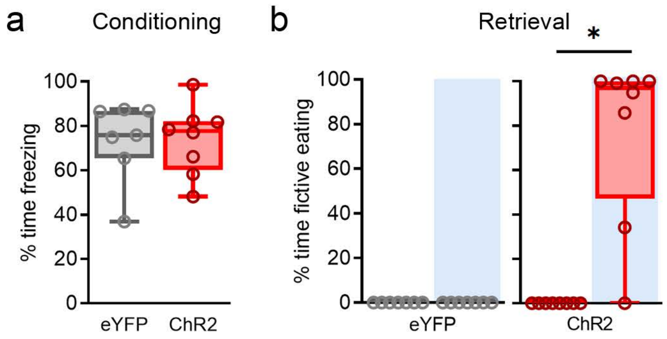

Supplementary Figure 6. Expression of conditioning-related freezing and fictive eating for BF^{GAD2+}→PAG optogenetic experiment. Related to Figure 5. **a**, Freezing levels during final two CS trials of conditioning. ChR2, n = 8; eYFP, n =7. b, Fictive eating during $laser^{ON}$ vs. $laser^{OFF}$ CS trials. ChR2, Wilcoxon signed rank test, *W* = 28, * $p < 0.05$. ChR2, n = 8; eYFP, n = 7.
